# Investigating the effect of structural changes of two stretching disks on the dynamics of the MHD model

**DOI:** 10.1038/s41598-023-48988-4

**Published:** 2023-12-09

**Authors:** Ali Ahmadi Azar, Bahram Jalili, Payam Jalili, Davood Domiri Ganji

**Affiliations:** 1 Department of Mechanical Engineering, North Tehran Branch, Islamic Azad University, Tehran, Iran; 2 Department of Mechanical Engineering, Babol Noshirvani University of Technology, P.O. Box 484, Babol, Iran

**Keywords:** Mechanical engineering, Fluid dynamics

## Abstract

The purpose of this theoretical study is to explore the behavior of an electrically conducting micropolar fluid when subjected to a uniform magnetic field along the vertical axis between two stretching disks as the structure of the problem changes. In this context, structural changes refer to alterations in the distance between the two discs or the stretching rate of the two discs. The governing equations of this problem are a set of nonlinear coupled partial differential equations, which are transformed into a nonlinear coupled ordinary differential equation set by a similarity transformation. The transformation results in four dimensionless quantities and their derivatives that appear in the equations. Nine dimensionless parameters are derived via similarity variables, including stretching Reynolds number, magnetic parameter, radiation parameter, Prandtl number, Eckert number, Schmidt number, and three micropolar parameters. Previous similarity solutions focused on analyzing the effect of changes in each parameter on the four dimensionless quantities. However, this type of analysis is mainly mathematical and does not provide practical results. This study’s primary novelty is to redefine the magnetic parameter, Eckert number, stretching Reynolds number, and two micropolar parameters to analyze physical parameters that depend on the stretching rate of the two discs or the distance between them. The semi-analytical hybrid analytical and numerical method (HAN-method) is used to solve the equations. The results demonstrate that structural changes affect all five quantities of radial velocity, axial velocity, microrotation, temperature, and concentration. The study’s most significant finding is that an increase in the stretching rate of the two disks causes a sharp increase in temperature and Nusselt number. Conversely, increasing the distance between the two disks causes a sharp decrease in micro-rotation and wall couple stress. They were compared to a previous study in a specific case to validate the results’ accuracy.

## Introduction

### Importance and application of the problem

Investigating the problems related to the flow of a fluid by one or two disks that are stretching or rotating has received a lot of attention today, and due to the similar solutions of these problems, they have many applications such as rotary pumps, fans, turbines, boilers and chemical storage, cyclone separators and rotating disks of nuclear reactors, and have attracted considerable attention^1^.

### Literature review

The Von Kármán rotational viscous flow is a well-known classical problem in fluid mechanics, and Von Kármán^2^, in this famous problem, investigated the viscous fluid flow resulting from the rotation of a disk in such a way that the fluid far from the disk is stationary and introduced new variables called similarity transformations that allowed PDEs to be converted to ODEs, which later, called the Von Kármán similarity variables. More recent results were obtained by Cochran^3^ by modifying von Karman’s problem because Von Karman’s answers contained errors. The Von Kármán swirling fluid flow problem was solved more accurately later by Abdou^4^ with the homotopy perturbation method (HPM) and also by Yao and Lian^5^ with the Runge–Kutta method. The Von Karman rotational viscous flow problem, which was created by a rotating disk with an infinite radius, has the simplest physics among the problems related to fluid flow resulting from disks. Turkyilmazoglu^6^ improved the physics of Von Kármán’s rotating fluid problem, which Abdou studied^4^ and Yao and Lian^5^, and this time considered holes on the rotating disk that made the medium porous. Turkyilmazoglu^7^ improved the physics of the previous study^6^ by considering the magnetic field in the constitutive equations. The improvements of the Doh and Muthtamilselvan^8^ were in considering the micropolar fluid as the rotating fluid, and unlike other studies^2–7^, the considered constitutive equations were time-dependent. The obtained results showed that the Nusselt number increases with the increase of the instability parameter. Also, the results showed that as the magnetic parameter increases, the tangential velocity of microrotation decreases. The improvements of Sahoo et al.^9^ were in considering the non-Newtonian Reiner–Rivlin fluid as the rotating fluid, and unlike other studies^2–8^, the disk was stationary, and the fluid was rotated at infinity. Srivastava^10^ investigated the incompressible micropolar fluid between two rotating disks in a magnetic field. The most prominent novelty of this study was the use of two rotating discs instead of one rotating disc. Das and Sahoo^11^ improved the problem that was studied by Srivastava^10^ by considering the non-Newtonian Reiner–Rivlin fluid as the rotating fluid instead of regular micropolar fluid. Iqbal et al.^12^ improved the problems that were studied by Srivastava^10^ and Das and Sahoo^11^ by considering the transient constitutive governing equations, choosing electrically conducting incompressible water-based nanofluid instead of regular micropolar fluid and considering the porous medium for fluid flow. The structure of two rotating disks studied by Srivastava^10^, Das and Sahoo^11^, and Iqbal et al.^12^ was later improved by Jalili et al.^13^ and Agarwal^14^, and instead of two rotating disks, two stretching disks were used with the difference that Jalili et al.^13^ used the semi-analytical HAN method while Agarwal^14^ used HPM for solving the constitutive equations. As different methods were used, different results were concluded. The Agarwal^14^ results showed that the nature of the flow of radial velocity for different values of Reynolds number and the magnetic parameter is similar i.e. radial velocity decreases with an increase in both parameters near the central plane. The Jalili et al.^13^ results showed that The average error between HAN method and HPM, for radial velocity is 3.1%, for axial velocity is 0.69%. Akhter et al.^15^ improved the studies that applied by Jalili et al.^13^ and Agarwal^14^ with considering the porous medium for fluid flow. Hayat et al.^16^ used Jeffrey nanofluid between two rotating stretchable disks. Compared to previous studies^1–15^, this innovation was the simultaneous rotation and expansion of two disks and the use of Jeffrey’s non-Newtonian nano-fluid.

### Problem statement

This study shows the effect of structural changes of two coaxial stretching disks in the heat and mass transfer problem that fluid is micropolar, viscous, incompressible, and the fluid flow is steady, laminar, and axisymmetric. A uniform magnetic field along the z-axis is applied perpendicularly to the disks. So, the fluid flow model is MHD due to the electrically conducting fluid flow and the magnetic field. The governing equations of this problem are a set of nonlinear coupled PDEs. The similarity transformation is applied for converting the constitutive equations into a set of nonlinear coupled ODEs. The HAN method is used in this study due to its brilliant reputation for solving problems with nonlinear constitutive equations^13,17–24^. In the similarity solutions, five physical quantities such as radial velocity, axial velocity, micro-rotation, temperature, and concentration profiles, with the addition of nine physical parameters such as radiation parameter, Eckert number, Prandtl number, stretching Reynolds number, magnetic parameter, Schmidt number, vortex viscosity, micro-inertia density, and spin gradient viscosity parameter. The structural properties are the stretching rate of the two discs and the distance between the two discs. In this study, the changes in structural properties are investigated due to the effectiveness of these properties in all five mentioned quantities.

### Why the HAN method?

We chose the HAN method because of its mathematical process for reaching the solution. Many numerical techniques exist, but the main disadvantage of a numerical method is its lack of analytical solution. The HAN method has corrected this disadvantage of the numerical solutions in many problems^13,17–24^ and can also be applied in many recent numerical studies^25–35^. Understanding this method and reaching to analytical solutions is possible and quicker than other studies^36,37^ that have used other semi-analytical methods.

## Methodology

**Figure 1:**
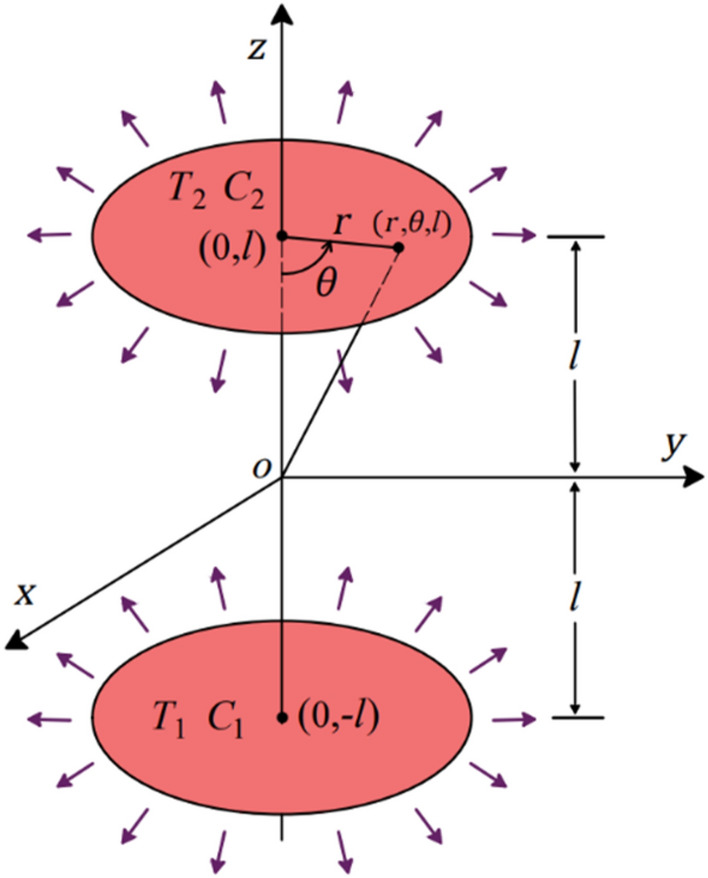
The schematic of the two stretchable disks.

The governing equations of the electrically conducting fluid are micropolar, viscous, and incompressible, and the fluid flow condition is steady, laminar, and axisymmetric. The governing equations related to this physics are explained in detail in the studies of Jalili et al.^13^ and Agarwal^14^. The fluid flow resulted from two stretchable disks with a uniform magnetic field along the z-axis. The geometry of the problem is demonstrated in Fig. 1 as follows:

The circular cylindrical coordinate system of $\left(r, \theta , z\right)$, is suitable for demonstrating the governing equations of mass, momentum, microrotation, energy, and concentration due to the schematic of the two disks. So, the constitutive equations via the similar variable of $\xi =z/l$ are as follows^13,14,38^:

1 $$ \frac{\partial u}{{\partial r}} + \frac{1}{l}\frac{\partial w}{{\partial \xi }} + \frac{u}{r} = 0, $$

2 $$ \rho \left( {u\frac{\partial u}{{\partial r}} + \frac{w}{l}\frac{\partial u}{{\partial \xi }}} \right)\begin{array}{*{20}c} \\ { = - \frac{\partial p}{{\partial r}} - \frac{\kappa }{l}\frac{{\partial N_{2} }}{\partial \xi } + \left( {\mu + \kappa } \right)\left( {\frac{{\partial^{2} u}}{{\partial r^{2} }} + + \frac{1}{{l^{2} }}\frac{{\partial^{2} u}}{{\partial \xi^{2} }} + \frac{1}{r}\frac{\partial u}{{\partial r}} - \frac{u}{{r^{2} }}} \right) - \sigma_{el} B_{os}^{2} u ,} \\ \end{array} $$

3 $$ \rho \left( {u\frac{\partial w}{{\partial r}} + \frac{w}{l}\frac{\partial w}{{\partial \xi }}} \right) = - \frac{1}{l}\frac{\partial p}{{\partial r}} + \kappa \left( {\frac{{\partial N_{2} }}{\partial r} + \frac{{N_{2} }}{r}} \right) + \left( {\mu + \kappa } \right)\left( {\frac{{\partial^{2} w}}{{\partial r^{2} }} + \frac{1}{{l^{2} }}\frac{{\partial^{2} w}}{{\partial \xi^{2} }} + \frac{1}{r}\frac{\partial w}{{\partial r}}} \right) , $$

4 $$ \rho j\left( {u\frac{{\partial N_{2} }}{\partial r} + \frac{w}{l}\frac{{\partial N_{2} }}{\partial \xi }} \right) = \kappa \left( {\frac{1}{l}\frac{\partial u}{{\partial \xi }} - \frac{\partial w}{{\partial r}}} \right) + \alpha_{3} \left( {\frac{{\partial^{2} N_{2} }}{{\partial r^{2} }} - \frac{{N_{2} }}{{r^{2} }} + \frac{1}{r}\frac{{\partial N_{2} }}{\partial r} + \frac{1}{{l^{2} }}\frac{{\partial^{2} N_{2} }}{{\partial \xi^{2} }}} \right) - 2\kappa N_{2} - \sigma_{el} B_{os}^{2} N_{2} , $$

5 $$ \rho c_{{\text{p}}} \left( {u\frac{\partial T}{{\partial r}} + \frac{w}{l}\frac{\partial T}{{\partial \xi }}} \right) - k\left( {\frac{1}{{l^{2} }}\frac{{\partial^{2} T}}{{\partial \xi^{2} }} + \frac{{\partial^{2} T}}{{\partial r^{2} }} + \frac{1}{r}\frac{\partial T}{{\partial r}}} \right) + \frac{1}{l}\frac{{\partial q_{rh} }}{\partial \xi } - \frac{\mu }{{l^{2} }}\left( {\frac{\partial u}{{\partial \xi }}} \right)^{2} - \sigma_{el} B_{os}^{2} \left( {u^{2} + N_{2}^{2} } \right) = 0, $$

6 $$ u\frac{\partial C}{{\partial r}} + \frac{w}{l}\frac{\partial C}{{\partial \xi }} = D_{{\text{e}}} \left( {\frac{{\partial^{2} C}}{{\partial r^{2} }} + \frac{1}{{l^{2} }}\frac{{\partial^{2} C}}{{\partial \xi^{2} }} + \frac{1}{r}\frac{\partial C}{{\partial r}}} \right) , $$

Where $u$ is the velocity in the direction of $r$, velocity in the direction of $z$ is denoted by $w$. The pressure field is denoted by $p$, dynamic viscosity is denoted by $\mu$, the vortex viscosity is denoted by $\kappa$, the micro inertia per unit mass is denoted by $j$,${ N}_{2}$ the microrotation velocity in the direction of $\theta$, fluid density is denoted by $\rho$, and ${\alpha }_{2}$, is the gyro-viscosity coefficient. ${\sigma }_{\mathrm{el}}$, is the electrical conductivity of the fluid, $T$ the temperature scalar field, ${c}_{\mathrm{p}}$ is the specific heat capacity at constant pressure, $k$ the coefficient of thermal conductivity,${q}_{\mathrm{rh}}$ is the radiation heat flux,$C$ is the fluid concentration, ${D}_{\mathrm{e}}$ , is the diffusion coefficient. Where $k$ is the thermal conduction, ${B}_{\mathrm{os}}$ the strength of the magnetic field and the radiation heat flux ${q}_{\mathrm{rh}}$ is demonstrated in Eq. (7) as follows^39^:

7 $$ q_{{{\text{rh}}}} = - \frac{4\sigma }{{3k_{{\text{a}}} }}\frac{{\partial \left( {T^{4} } \right)}}{\partial z} = - \frac{4\sigma }{{3lk_{{\text{a}}} }}\frac{{\partial \left( {T^{4} } \right)}}{\partial \xi } , $$

Here, $\sigma$ is the Stefan-Boltzmann constant and ${k}_{a}$, the average absorption coefficient. By defining Eq. (7), and substituting this equation into Eq. (5), the ${q}_{\mathrm{rh}}$ parameter in Eq. (5) gives his place to the right side of Eq. (7). The positions of the disks are in $z=l$ and $=-l$ . The boundary conditions related to the physics of the problem in Eqs. (1–6) are as follows^13,14^:

8 $$ \left\{ { \begin{array}{*{20}l} {u = rS,\quad w = 0,\quad N_{2} = 0,\quad T = T_{1} ,\quad C = C_{1} ,\quad when\quad z = - l,} \hfill \\ {u = rS,\quad w = 0,\quad N_{2} = 0,\quad T = T_{2} ,\quad C = C_{2} ,\quad when\quad z = l,} \hfill \\ \end{array} } \right. $$

Here, $S$ is the stretching parameter of the disks, $T_{1}$ and $T_{2}$ the temperature of the lower and upper disks, respectively, $C_{1}$ and $C_{2}$ the fluid concentration on the lower and upper discs, respectively. The governing equations of the problem in Eqs. (1–6) will be transformed from the form of the PDEs to the ODEs by the following similarity transformations:

9 $$ \begin{array}{*{20}l} {u = - \frac{rS}{2}f^{\prime}\left( \xi \right) ,\quad w = Slf\left( \xi \right),\quad N_{2} = - \frac{rS}{{2l^{2} }}g\left( {\upxi } \right), } \hfill \\ {\theta \left( \xi \right) = \frac{{T - T_{2} }}{{T_{1} - T_{2} }},\quad \phi \left( \xi \right) = \frac{{C - C_{2} }}{{C_{1} - C_{2} }},} \hfill \\ \end{array} $$

where $\theta (\xi )$ and $\phi \left(\xi \right)$ are the dimensionless temperature and concentration, respectively, $f(\xi )$ is the axial velocity, $f{\prime} (\xi )$ is the radial velocity, and $g(\xi )$ is the microrotation profile. By using similarity transformations of Eq. (9), the system of PDEs in Eqs. (1–6) can be converted into the following system of nonlinear ODEs:

10 $$ F: = \left( {1 + \lambda_{1} } \right)f^{\left( 4 \right)} - \lambda_{1} g^{\prime\prime} - R_{0} ff^{\prime\prime\prime} - R_{0} M_{n}^{2} f^{\prime\prime} = 0, $$

11 $$ G: = \lambda_{3} g^{\prime \prime } + \lambda_{1} \left( {f^{\prime \prime } - 2 g} \right) + R_{0} \lambda_{2} \left( {\frac{1}{2}f^{\prime } g - fg^{\prime } } \right) = 0, $$

12 $$ \Theta :{\text{ = }}\left( {1 + \frac{4}{3}N_{r} } \right)\theta ^{{\prime \prime }}  + \frac{1}{4}P_{r} E_{c} \left( {f^{{\prime \prime }} } \right)^{2}  - R_{0} P_{r} f\theta ^{\prime }  = 0, $$

13 $$ \Phi :{\text{ = }}\phi ^{{\prime \prime }}  + R_{0} S_{c} f\phi ^{\prime }  = 0,$$

here, $R_{0} = \rho Sl^{2} /\mu$ is the stretching Reynolds number, $M_{{\text{n}}} = \left( {\sigma_{{{\text{el}}}} B_{{{\text{os}}}}^{2} /\rho S} \right)^{1/2}$ is the magnetic parameter, $\lambda_{1} = \kappa /\mu$ is the vortex viscosity, $\lambda_{2} = j/l^{2}$ is the micro-inertial density, $\lambda_{3} = \alpha_{3} /\mu l^{2}$ is the spin gradient viscosity parameter, $P_{{\text{r}}} = \mu c_{{\text{p}}} /k$ is the Prandtl number, $N_{{\text{r}}} = 4\sigma T_{2}^{3} /k_{{\text{a}}} k$ is the radiation parameter, $E_{{\text{c}}} = {\text{U}}^{2} /\left( {T_{1} - T_{2} } \right)c_{{\text{p}}} = r^{2} S^{2} /\left( {T_{1} - T_{2} } \right)c_{{\text{p}}}$ is the Eckert number, $Sc = \nu /D_{{\text{e}}}$ is the Schmidt number^13,14^.

The similarity transformations of Eq. (9) also affect the boundary conditions in Eq. (8) and turn it into the following form:

14 $$ \left\{ {\begin{array}{*{20}l} {f = 0 ,\quad f^{\prime } = - 2,\quad g = 0,\quad \theta = 1,\quad \phi = 1\quad when\quad \xi = - 1,} \hfill \\ {f = 0,\quad f^{\prime } = - 2,\quad g = 0,\quad \theta = 0,\quad \phi = 0\quad when\quad \xi = 1,} \hfill \\ \end{array} } \right. $$

the values ​​of the Nusselt number ($N_{{\text{u}}}$), skin friction coefficient ($C_{{\text{f}}}$), and wall couple stress ($C_{{\text{g}}}$) can be calculated for the upper and lower disks as follows:

15 $$  C_{{\text{f}}}  =  - \frac{{\left( {1 + \lambda _{1} } \right)f^{{\prime \prime }} \left( { \pm 1} \right)}}{{2R_{{\text{e}}} }},\quad C_{{\text{g}}}  = \frac{{\lambda _{3} }}{{2R_{{\text{e}}} }}{\text{g}}^{\prime } \left( { \pm 1} \right),\quad N_{{\text{u}}}  =  - \theta ^{\prime } \left( { \pm 1} \right),    $$

where in Eq. (15), $R_{{\text{e}}} = \rho Slr/\mu$ is the local Reynolds number, $S$, the stretching parameter of the disks, $l$ the distance from $r$-axis, $\mu$ the dynamic viscosity, and $r$ an arbitrary radius of the disk. It can be seen in the local Reynolds number and Eckert number there is a parameter of the radius of the disk, and in problems that deal with stretchable disks, the radius of the disks is not counted in the structural parameter because the radius of the disks assumed infinity.

## Application of the HAN method

The HAN method was applied in several studies^13,17–24^ for solving nonlinear ordinary differential equations, and its methodology was given in detail. We have used this method due to its flexibility advantage compared to other semi-analytical methods. The word “flexibility” is for this reason: many numerical methods can handle solving nonlinear differential equations, and when one numerical method doesn’t give us an answer, the other numerical methods do. And this is why it is so-called flexible. Another advantage of this method is that it is analytical and gives a more accurate analytical solution with fewer terms than other semi-analytical methods. To use the HAN method to obtain the semi-analytical solution of the system of ordinary differential equations of Eqs. (10–13), we first assume the four polynomials with constant coefficients as analytical solutions when $M_{n} = 2.5$, $\lambda_{1} = 2.0$, $\lambda_{2} = 0.2$, $\lambda_{3} = 0.3$, $P_{r} = 2.0$, $N_{r} = 1.0$, $E_{c} = 0.3$, $R_{0} = 20$, and $S_{C} = 0.5$ are as follows:

16 $$ f\left( \xi \right) = \mathop \sum \limits_{i = 0}^{12} a_{i} \xi^{i} ,\quad g\left( \xi \right) = \mathop \sum \limits_{i = 0}^{10} b_{i} \xi^{i} ,\quad {\uptheta }\left( \xi \right) = \mathop \sum \limits_{i = 0}^{10} c_{i} \xi^{i} ,\quad \phi \left( \xi \right) = \mathop \sum \limits_{i = 0}^{10} d_{i} \xi^{i} , $$

As can be seen in Eq. (16), there are 46 unknown coefficients, and by making 46 algebraic equations, these coefficients can be determined. The boundary conditions of the problem in Eq. (14) can make ten equations as follows:

17 $$ f\left( { - 1} \right) = \mathop \sum \limits_{i = 0}^{12} a_{i} \left( { - 1} \right)^{i} = 0, $$

18 $$ f^{\prime}\left( { - 1} \right) = \mathop \sum \limits_{i = 1}^{12} ia_{i} \left( { - 1} \right)^{i - 1} = - 2, $$

19 $$ g\left( { - 1} \right) = \mathop \sum \limits_{i = 0}^{10} b_{i} \left( { - 1} \right)^{i} = 0, $$

20 $$ \theta \left( { - 1} \right) = \mathop \sum \limits_{i = 0}^{10} c_{i} \left( { - 1} \right)^{i} = 1, $$

21 $$ \phi \left( { - 1} \right) = \mathop \sum \limits_{i = 0}^{10} d_{i} \left( { - 1} \right)^{i} = 1, $$

22 $$ f\left( 1 \right) = \mathop \sum \limits_{i = 0}^{12} a_{i} \left( 1 \right)^{i} = 0, $$

23 $$ f^{\prime}\left( 1 \right) = \mathop \sum \limits_{i = 1}^{12} ia_{i} \left( 1 \right)^{i - 1} = - 2, $$

24 $$ g\left( 1 \right) = \mathop \sum \limits_{i = 0}^{10} b_{i} \left( 1 \right)^{i} = 0, $$

25 $$ \theta \left( 1 \right) = \mathop \sum \limits_{i = 0}^{10} c_{i} \left( 1 \right)^{i} = , $$

26 $$ \phi \left( 1 \right) = \mathop \sum \limits_{i = 0}^{10} d_{i} \left( 1 \right)^{i} = 0, $$

Equations (17–26) are not enough to determine all coefficients in Eq. (16). So the rest of the remaining equations can be made from the numerical solution of Eqs. (10–13) with boundary conditions of Eq. (14) and Table 1 demonstrate the numerical solution.

**Table 1:** The numerical solution to the problem using the Runge–Kutta method.

$\xi$	$f\left(\xi \right)$	$g\left(\xi \right)$	$\uptheta \left(\xi \right)$	$\upphi \left(\xi \right)$
− 0.8	− 0.185528304641055	0.661457140985974	0.875452591504731	0.949646021377983
− 0.6	− 0.179888341075159	0.426732669027955	0.755221087287952	0.878529419214726
− 0.4	− 0.127806662413904	0.211368685666526	0.686797468355960	0.777536891871878
− 0.2	− 0.0650206162621988	0.0840754288323933	0.642657815202979	0.647660326445521
0	0	0	0.607452350449235	0.499999999999616
0.2	0.0650206162636657	− 0.0840754288494122	0.572246287576256	0.352339673555227
0.4	0.127806662413351	− 0.211368685665728	0.528079484460573	0.222463108128387
0.6	0.179888341075015	− 0.426732669029823	0.459011859259901	0.121470580785461
0.8	0.185528304641006	− 0.661457140987003	0.326038498252382	0.0503539786220983

As numerical solutions are approximated, we can consider them as approximated boundary conditions. As the problem’s boundary conditions make ten algebraic equations, the approximated boundary conditions of Table 1 can also make the rest of the equations. After constructing 46 algebraic equations, 46 unknown coefficients in Eq. (14) are known, and the semi-analytical solutions of differential equations of Eqs. (10–13) are as follows:

27 $$ \begin{aligned} f\left( \xi \right) & = - 3.720211699 \times 10^{ - 9} \xi^{12} - 0.04091768971 \xi^{11} + 1.184358446 \\ & \quad \times 10^{ - 8} \xi^{10} - 0.07734411475 \xi^{9} - 1.408300399 \times 10^{ - 8} \xi^{8} \\ & \quad - 0.09550809899 \xi^{7} + 7.689789586 \times 10^{ - 9} \xi^{6} - 0.08701266394 \xi^{5} \\ & \quad - 1.907780166 \times 10^{ - 9} \xi^{4} - 0.02548546760 \xi^{3} + 1.815554155 \\ & \quad \times 10^{ - 10} \xi^{2} + 0.3262680350 \xi - 3.933609945 \times 10^{ - 12} , \\ \end{aligned} $$

28 $$ \begin{aligned} g\left( \xi \right) & = - 3.158131846 \times 10^{ - 8} \xi^{10} + 2.068973197 \xi^{9} + 6.898922975 \times 10^{ - 8} \xi^{8} \\ & \quad - 0.6717361269 \xi^{7} - 5.065498752 \times 10^{ - 8} \xi^{6} - 0.1530302276 \xi^{5} \\ & \quad + 1.473356293 \times 10^{ - 8} \xi^{4} - 0.8584502512 \xi^{3} - 1.518200104 \\ & \quad \times 10^{ - 9} \xi^{2} - 0.3857565913 \xi + 3.171339418 \times 10^{ - 11} , \\ \end{aligned} $$

29 $$ \begin{aligned} \theta \left( \xi \right) & = - 0.2900875313 \xi^{10} + 0.1583540872 \xi^{9} + 0.2630140084 \xi^{8} \\ & \quad - 0.2555097202 \xi^{7} - 0.09021892852 \xi^{6} - 0.06857259657 \xi^{5} \\ & \quad + 0.01012428898 \xi^{4} - 0.1649673070 \xi^{3} - 0.0002841880364 \xi^{2} \\ & \quad - 0.1693044634 \xi + 0.6074523504, \\ \end{aligned} $$

30 $$ \begin{aligned} \phi \left( \xi \right) = & 6.180213242 \times 10^{ - 10} \xi^{10} - 0.01662615215 \xi^{9} - 1.349678161 \times 10^{ - 9} \xi^{8} \\ & \quad + 0.07046257333 \xi^{7} + 9.901703559 \times 10^{ - 10} \xi^{6} - 0.2095976314 \xi^{5} \\ & \quad - 2.870625460 \times 10^{ - 10} \xi^{4} + 0.4101374513 \xi^{3} + 2.893302654 \\ & \quad \times 10^{ - 11} \xi^{2} - 0.7543762411 \xi + 0.5000000000, \\ \end{aligned} $$

**Figure 2:**
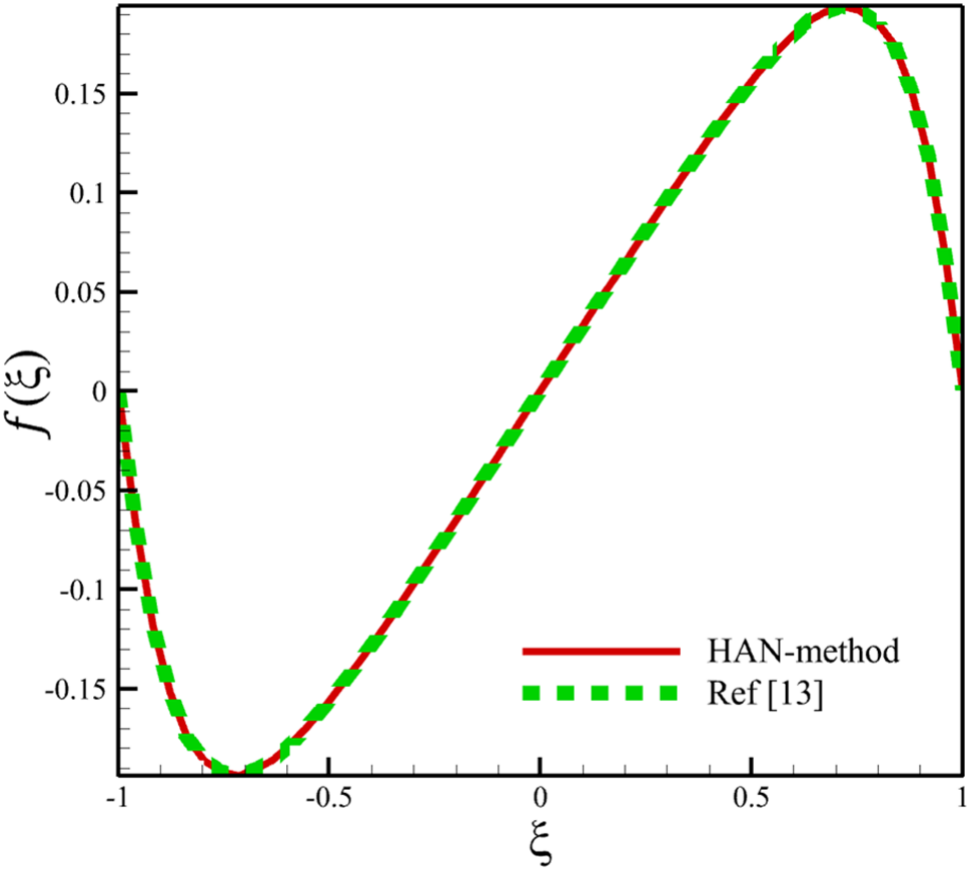
The comparison of axial velocity results.

**Figure 3:**
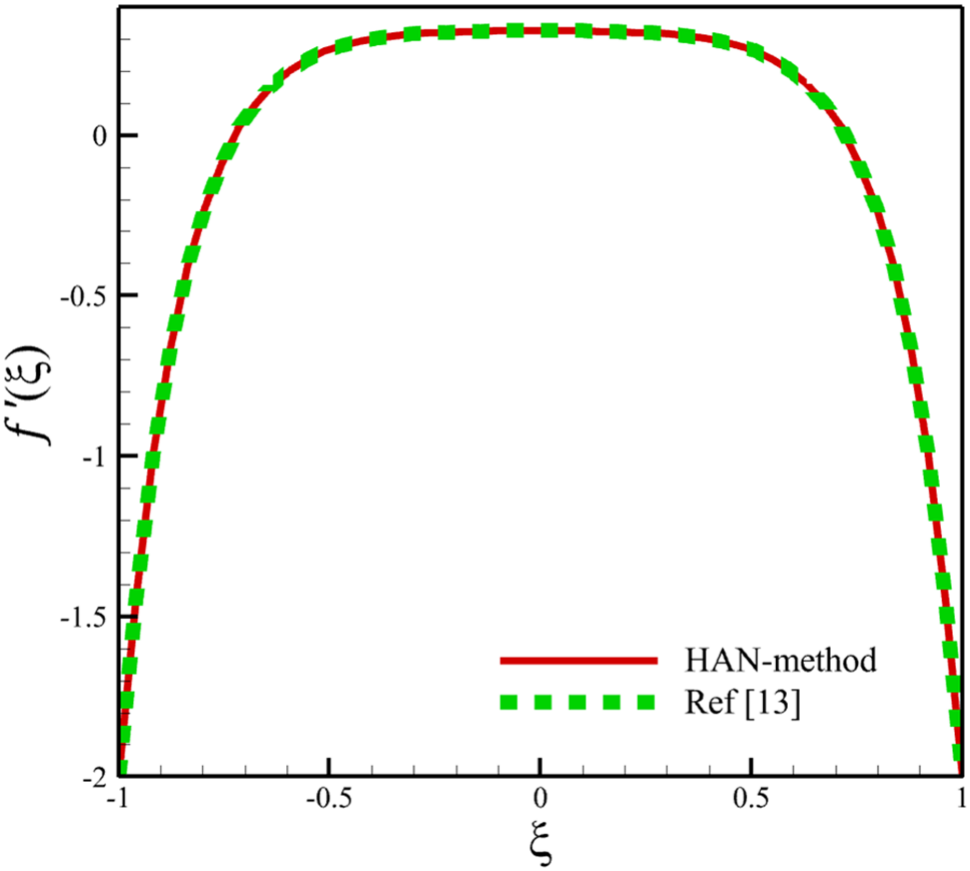
The comparison of radial velocity results.

**Figure 4:**
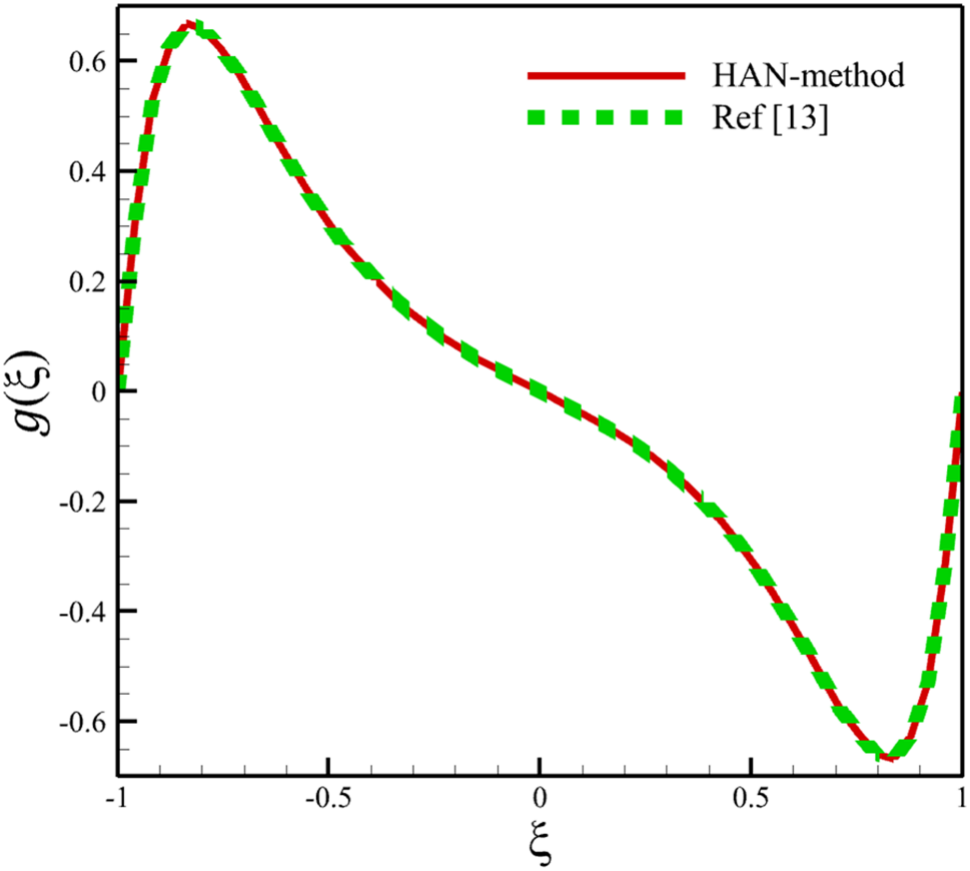
The comparison of micro-rotation velocity results.

**Figure 5:**
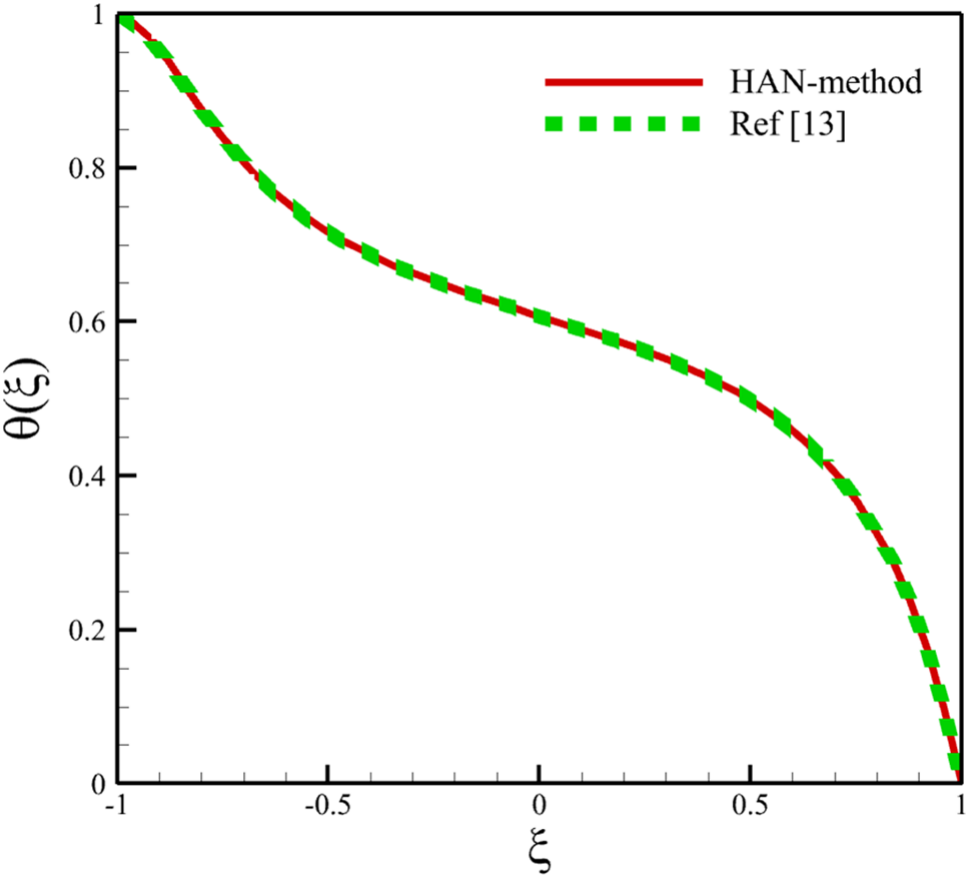
The comparison of temperature results.

**Figure 6:**
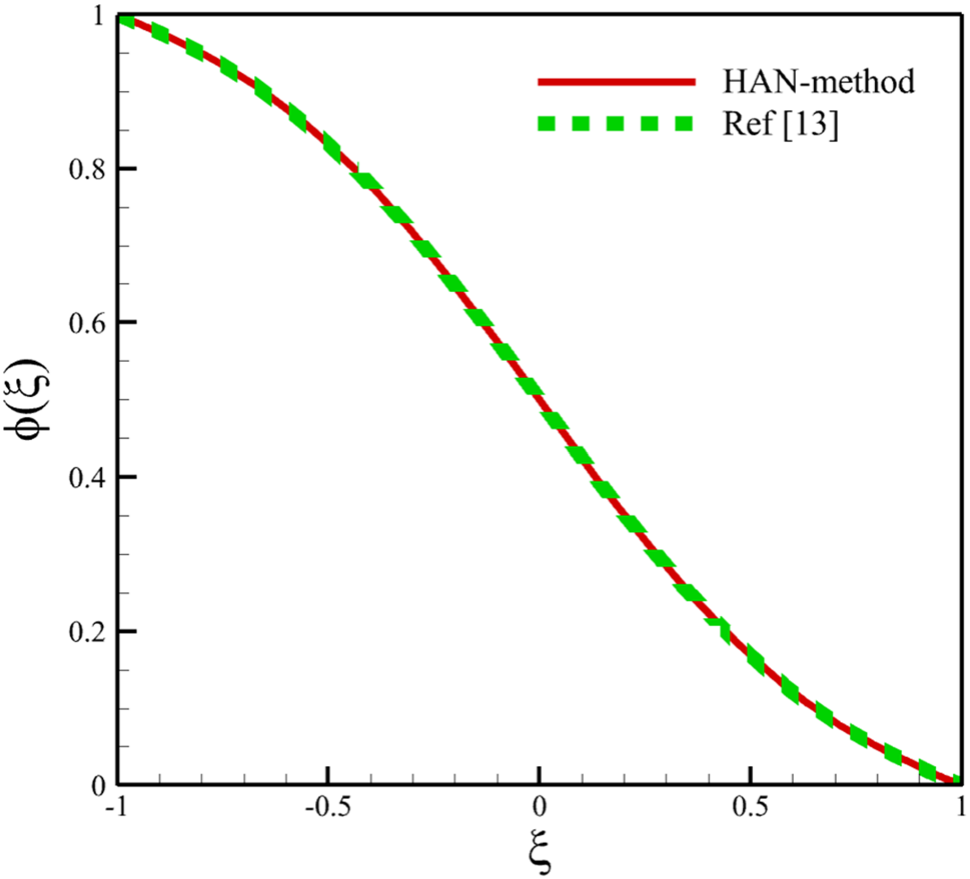
The comparison of concentration results.

Comparing analytical solutions of Eqs. (27–30) with other similar works, the validity of the current results will be proved. Comparison of the analytical solutions of the Eqs. (27–30) with reference^13^ are existed in Figs. 2, 3, 4, 5 and 6 as follows:

## Results and discussion

### Redefinition of the parameters

In this part, some physical parameters that depend on the stretching rate of the two disks or the distance between the two disks, including the magnetic parameter, Eckert number, stretching Reynolds number, and two of the micropolar parameters, are redefined. This redefinition is a reason to investigate the effect of structural changes on the physical quantities of the problem. After redefining the parameters, some of the parameters that depended on structural changes were written in terms of $S$ and $l$ parameters. Other parameters unrelated to the structure of the two discs were written as numbers, and $S$ and $l$ parameters are not seen in them. So, the redefined physical parameters are appointed as $M_{n} = \left( S \right)^{ - 1}$, $\lambda_{1} = 2.0$, $\lambda_{2} = l^{2}$, $\lambda_{3} = 0.3 l^{2}$, $P_{r} = 1.0$, $N_{r} = 1.0$, $E_{c} = S^{2}$, $R_{0} = Sl^{2}$, and $S_{C} = 0.5$.

### Effect of the stretching rate of the discs

**Figure 7:**
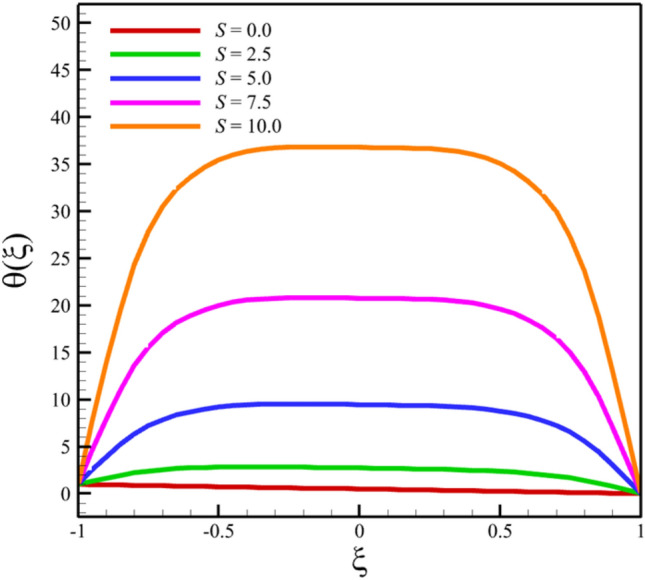
The effect of the stretching rate of both disks on the temperature profile.

**Figure 8:**
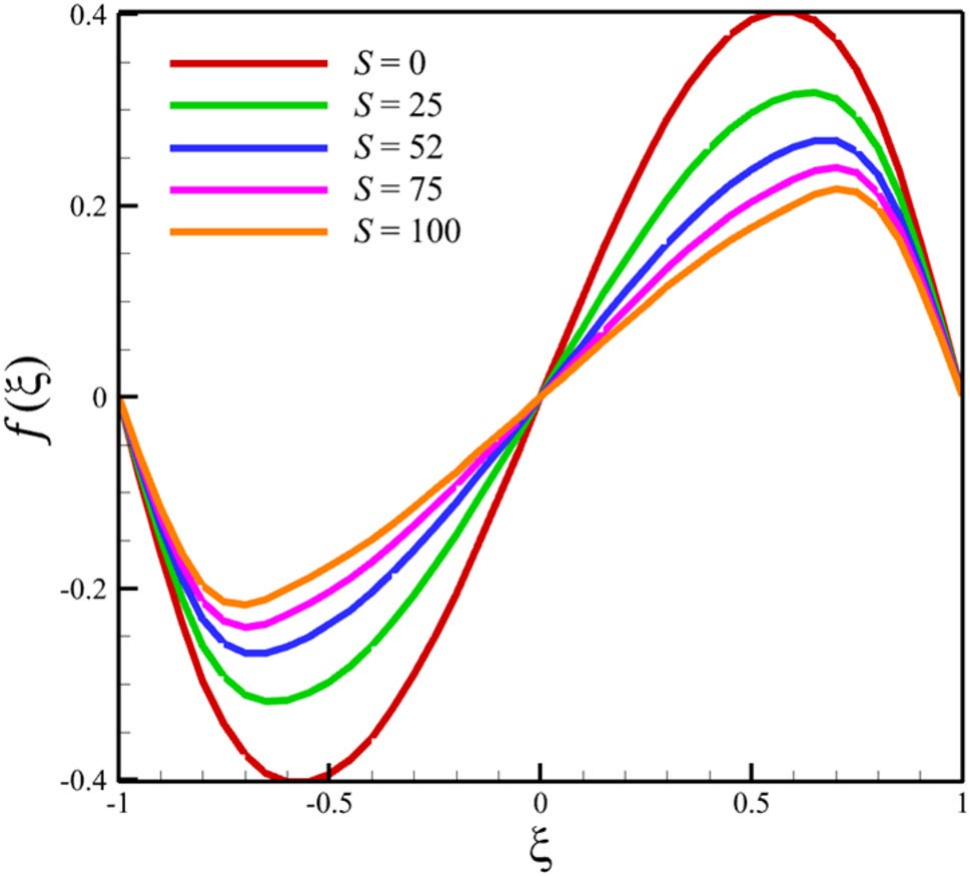
The effect of the stretching rate of both disks on the axial velocity profile.

**Figure 9:**
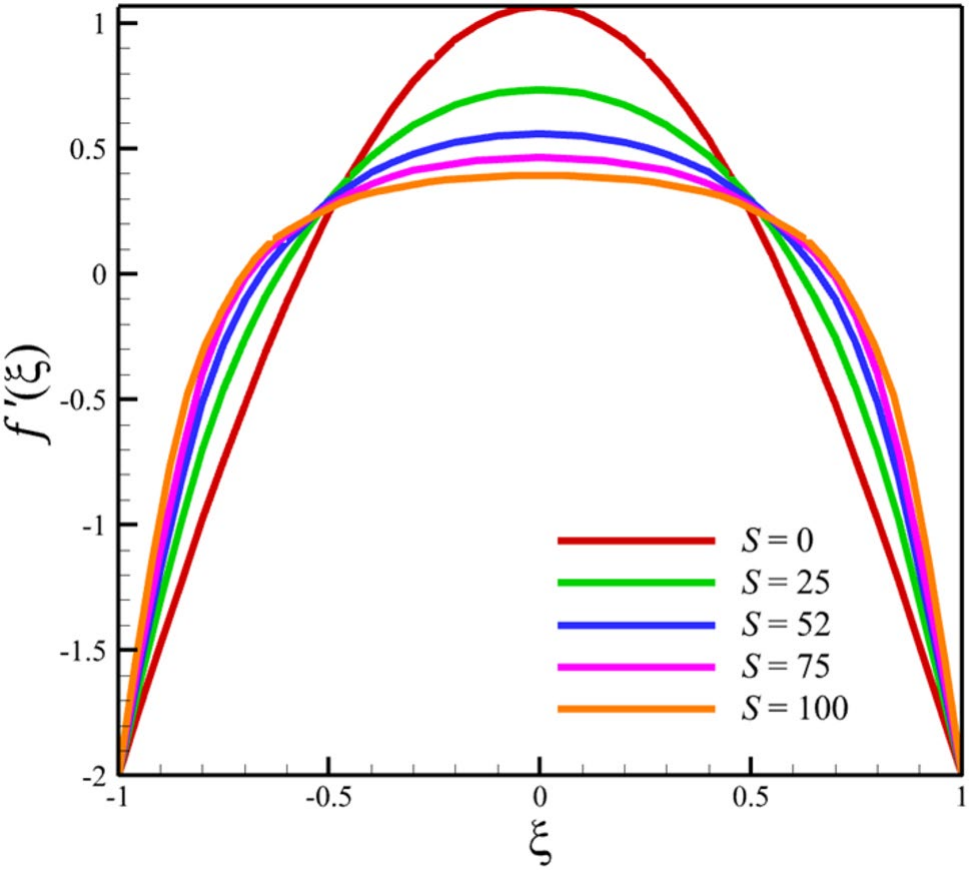
The effect of the stretching rate of both disks on the radial velocity profile.

**Figure 10:**
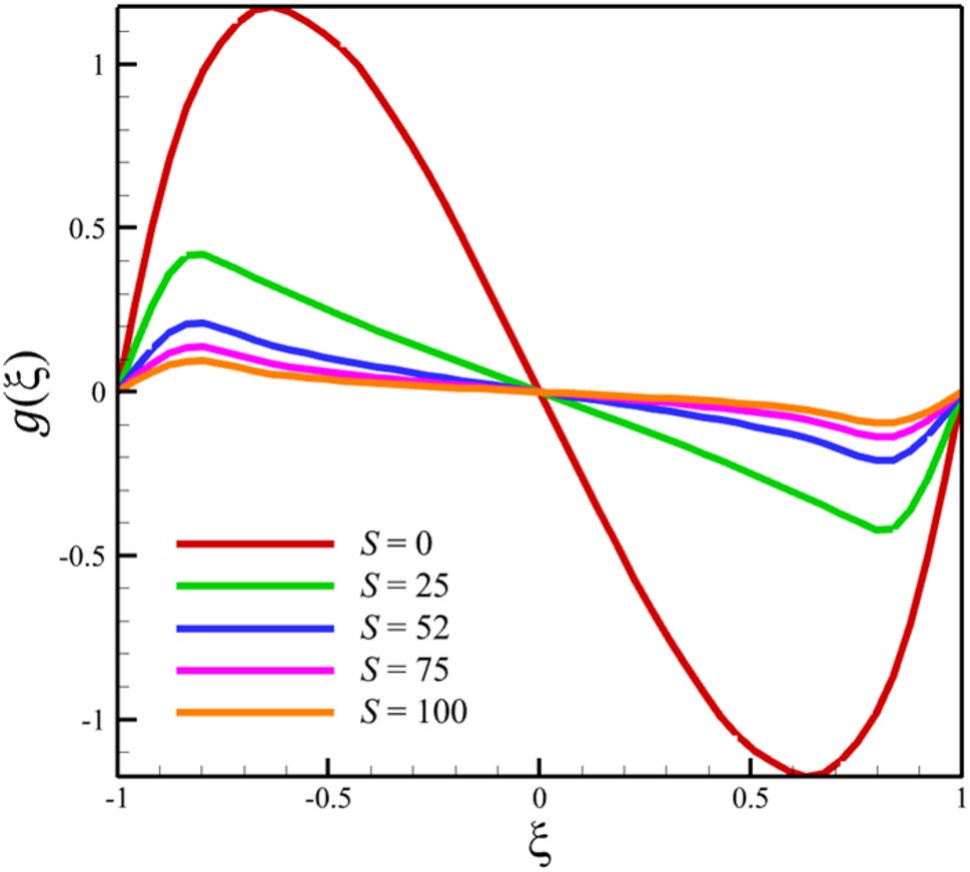
The effect of the stretching rate of both disks on the micro-rotation velocity profile.

**Figure 11:**
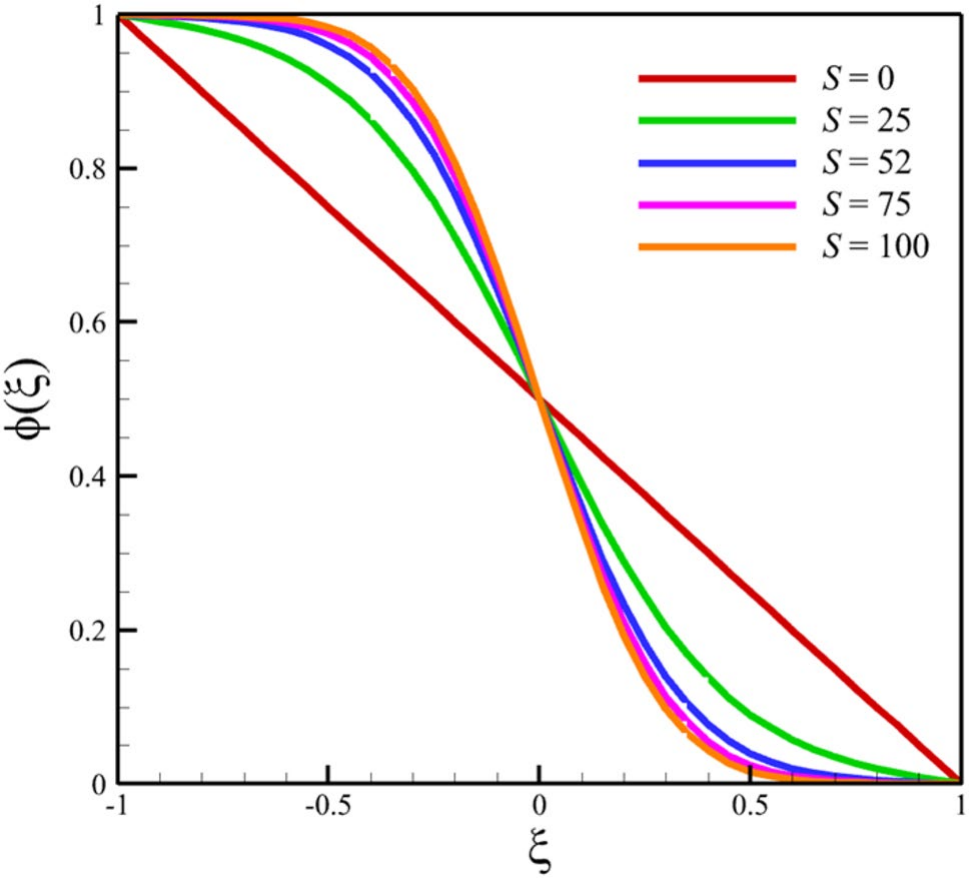
The effect of the stretching rate of both disks on the concentration profile.

Among the structural parameters, when the stretching rate of the two disks changes, the temperature profile will change the most, while the profiles of velocity, micro-rotation, and concentration do not change significantly when the stretching rate of the two disks changes. This high sensitivity of the temperature contour to the stretching rate of the two disks is shown in Fig. 7. That is, when the parameter $S$ changes from 0 to 10 and $l = 1$, the temperature quantity changes significantly, according to Figs. 8, 9, 10 and 11, the quantities of axial velocity, microrotation, radial, and concentration change significantly when the value of $S$ changes from 0 to 100 and $l = 1$. In Fig. 7, as the stretching rate of the two disks is 10, the maximum temperature is 36.8238853633 and occurred where $\xi = - 0.15$. In Fig. 8, the maximum positive axial velocity decreases from 0.402416382596 to 0.217497070428 when the stretching rate of the two disks increases from 0 to 100. In Fig. 8, the maximum negative axial velocity decreases from − 0.402416382596 to − 0.217497070428 when the stretching rate of the two disks increases from 0 to 100. As the stretching rate of the two disks increases, the location where positive axial velocity occurs gets closer to the top disk, and the location where negative axial velocity occurs gets closer to the bottom disk. In Fig. 9, the maximum positive radial velocity decreases from 1.06737465069 to 0.393436047607 when the stretching rate of the two disks increases from 0 to 100. In Fig. 10, the maximum positive micro-rotation velocity decreases from 1.17621364281 to 0.0940631287495, and the maximum negative micro-rotation velocity decreases from − 1.17621364281 to − 0.0940631287496 when the stretching rate of the two disks increases from 0 to 100. In Fig. 11, increasing the stretching rate of the two disks will reduce the changes in concentration near the two disks.

**Figure 12:**
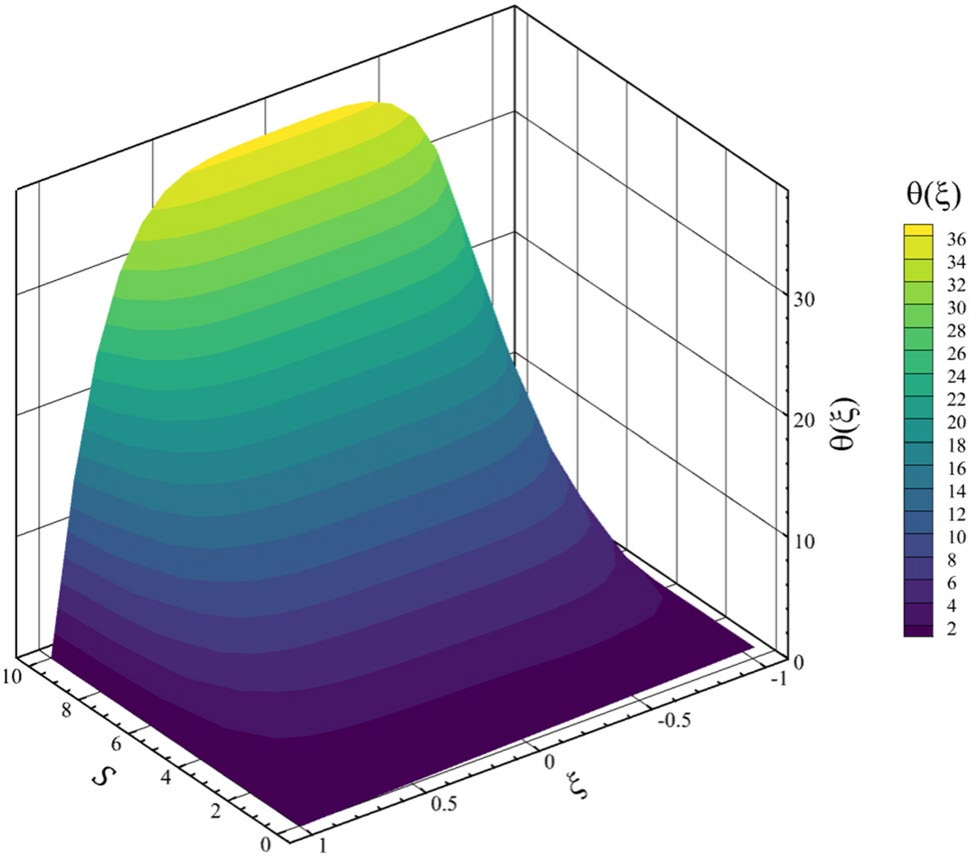
The 3D temperature contour when the stretching rate of both disks changes from 0 to 10.

**Figure 13:**
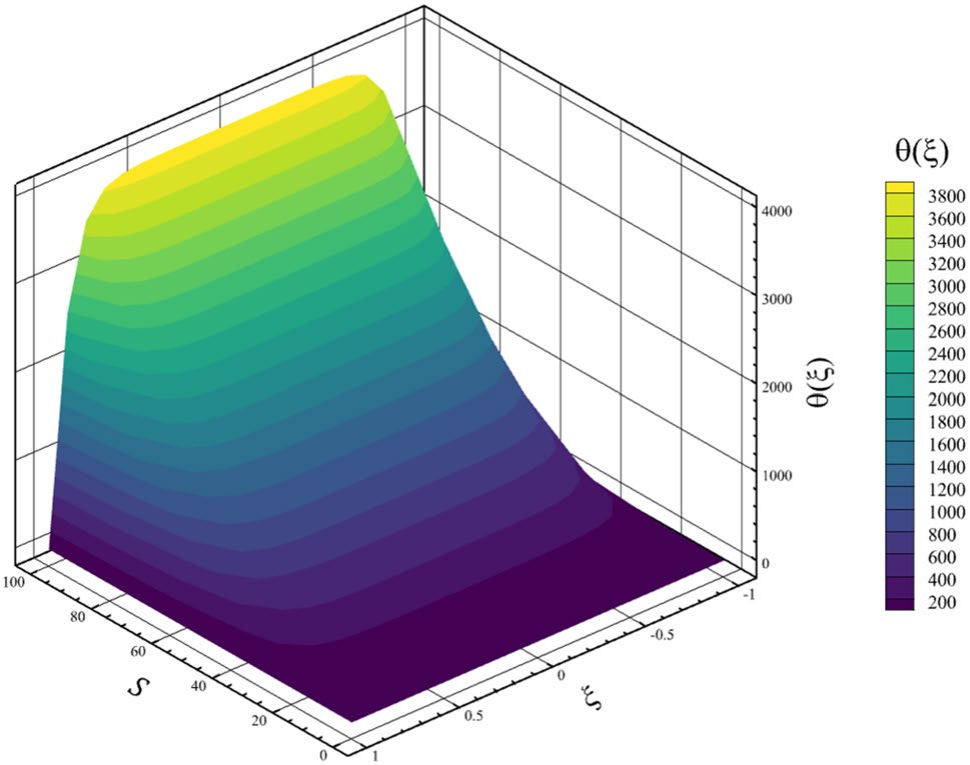
The 3D temperature contour when the stretching rate of both disks changes from 0 to 100.

**Figure 14:**
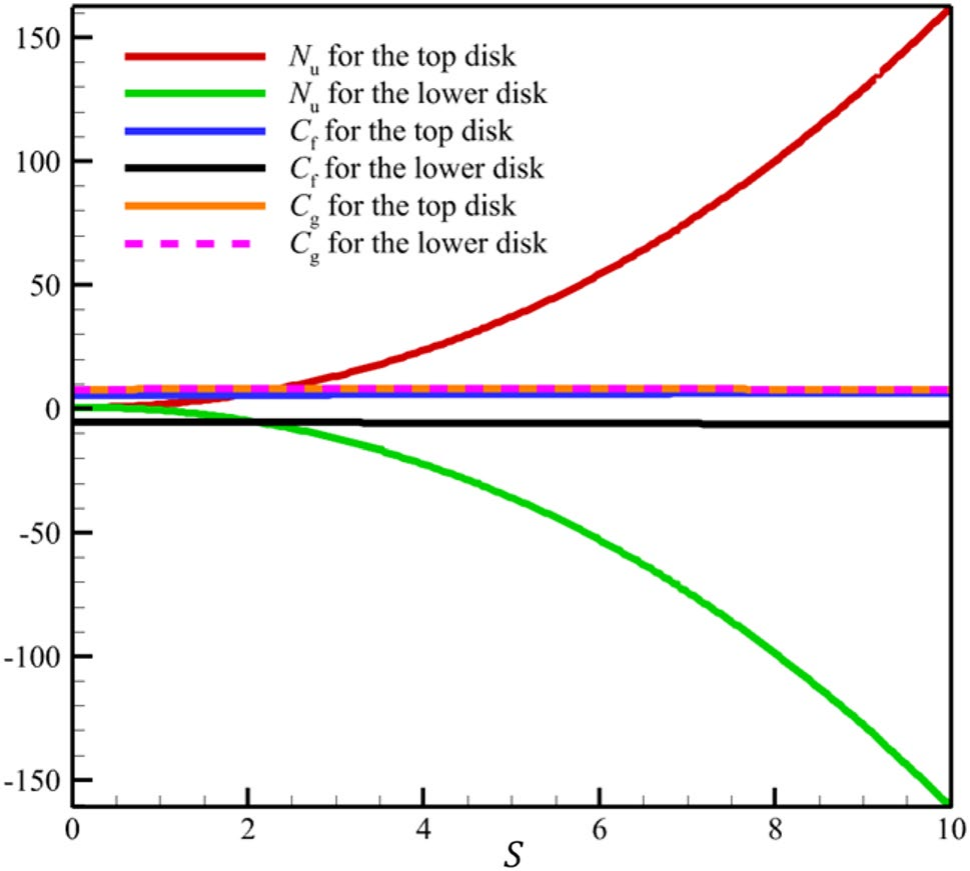
The variations of the Nusselt number (${N}_{\mathrm{u}}$), skin friction coefficient (${C}_{\mathrm{f}}$), and wall couple stress (${C}_{\mathrm{g}}$) with increasing stretching rates of two disks.

**Figure 15:**
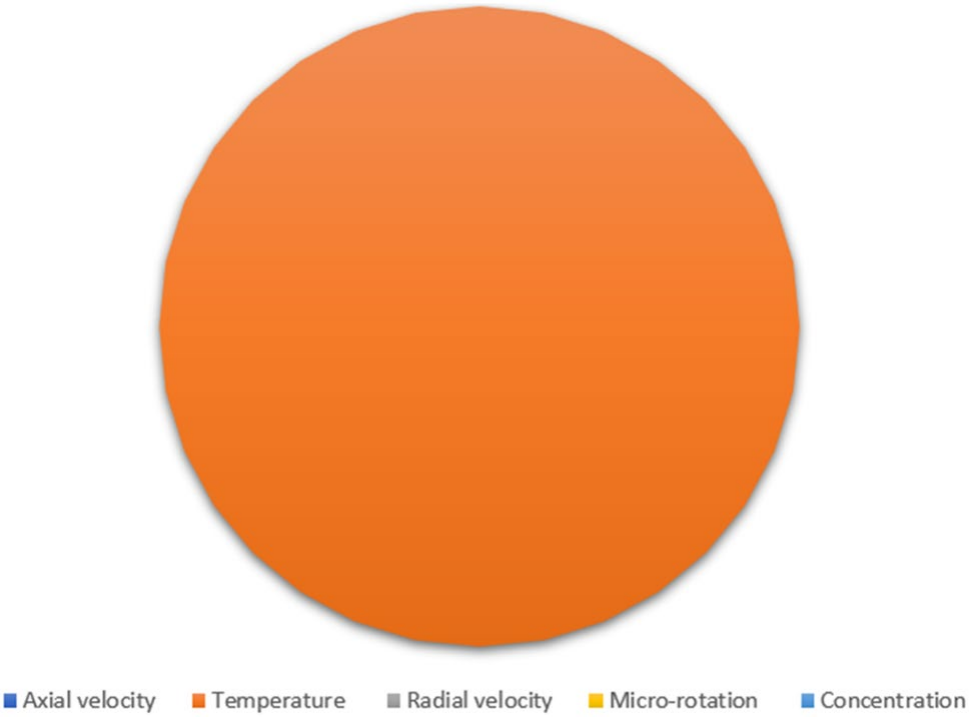
The contribution of the increase of each quantity by the increasing of the stretching rate of the discs when the parameter $S$ changes from 0 to 10 and $l=1$.

In continuation of the physical argument quantitatively, statistical analysis can also check the effect of increasing the stretching rate of the two disks on each quantity. In the statistical analysis method, the absolute value of the average change of each quantity is checked by the stretching rate of the two disks, and the contribution of each quantity change is shown in a pie chart. In Figs. 12 and 13, the three-dimensional temperature contours show the sensitivity of the temperature contour on the stretching rate of the two disks. According to Fig. 14, the Nusselt number for both disks is the most sensitive to the change in the stretching rate of the two disks, and according to Eq. (15), this reason for the sensitivity of the Nusselt number is due to the direct relationship of this number with temperature. According to Fig. 13, when the parameter $S$ changes from 0 to 100 and $l=1$, the average temperature value is changed from 0.5 to 3381.714411, which shows a 676242.8822% growth in fluid temperature. So, Fig. 15 shows the statistical analysis for the five quantities, and according to this figure, temperature has the biggest contribution, and the other quantities are almost nothing. A more detailed explanation of Fig. 15 can be that only the temperature changes significantly from the change in the stretching rate of the disks, and the contribution of other parameters from this dependence on the increase in the stretching rate is almost negligible.

**Figure 16:**
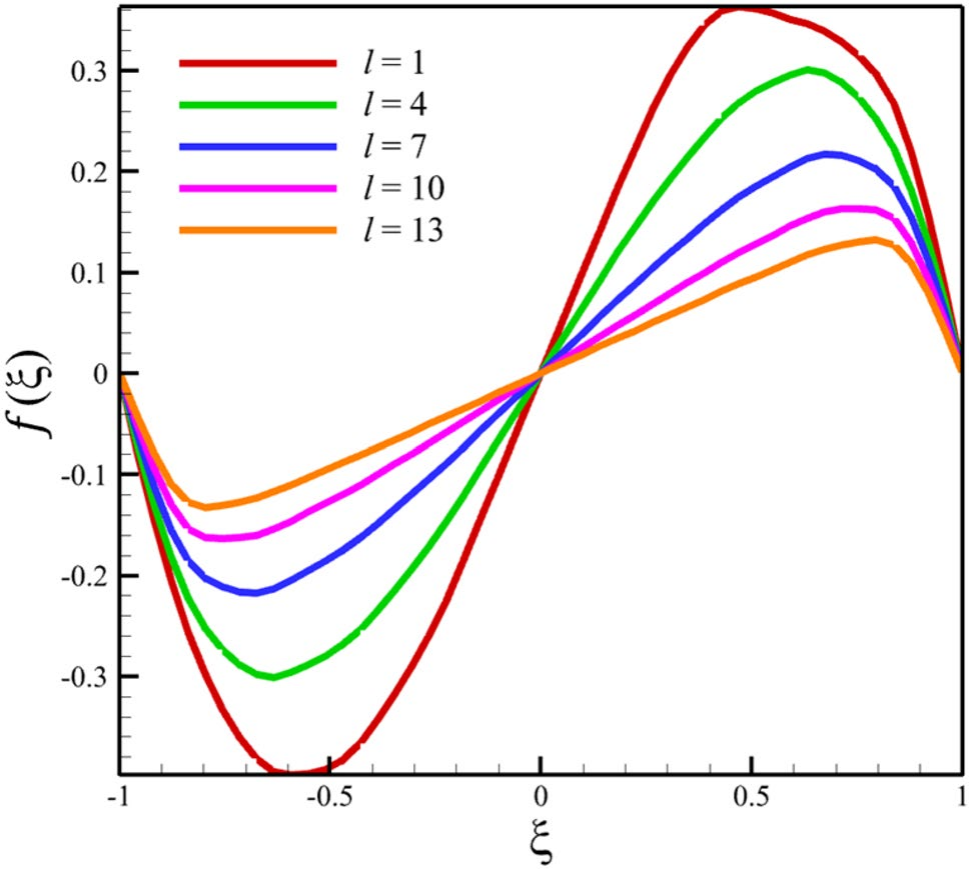
The effect of the distance between the two disks on the axial velocity profile.

**Figure 17:**
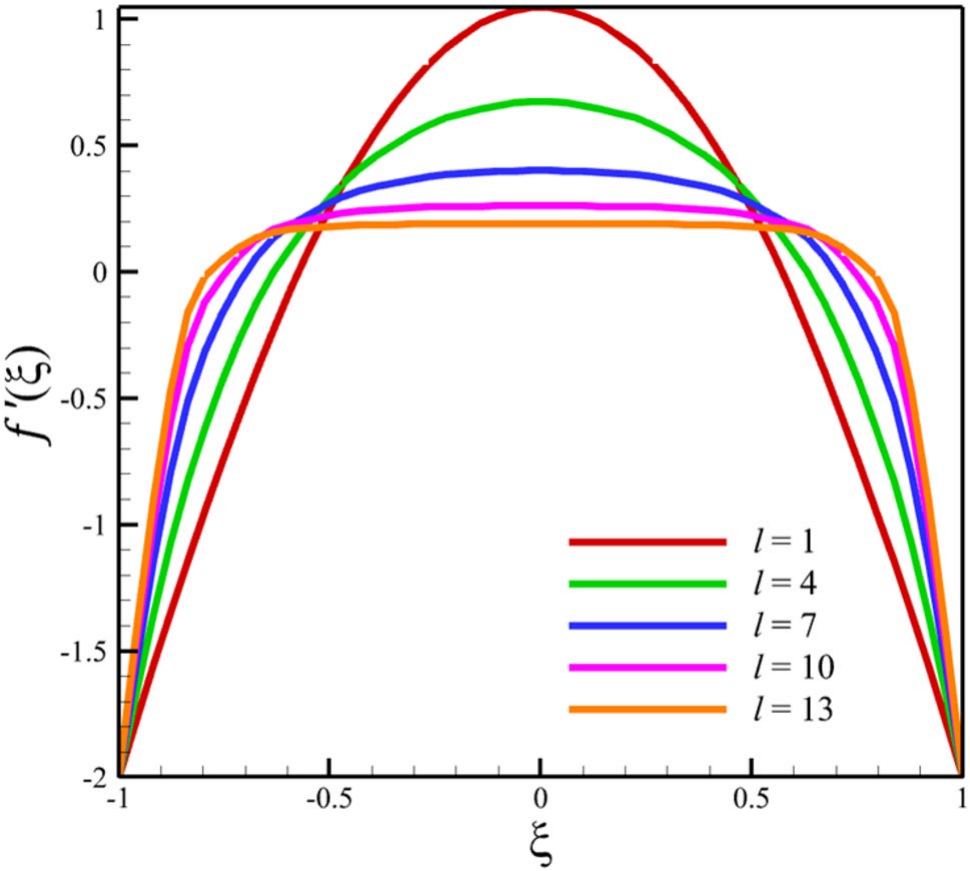
The effect of the distance between the two disks on the radial velocity profile.

### Effect of the distance between the two disks

**Figure 18:**
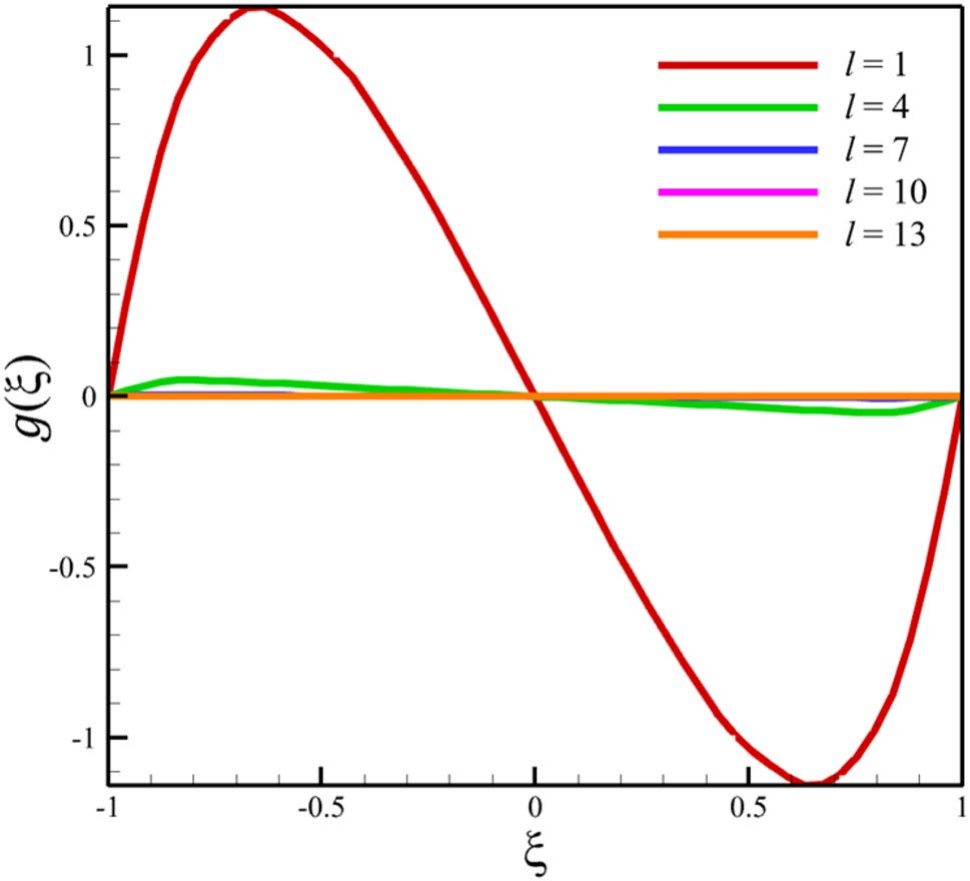
The effect of the distance between the two disks on the micro-rotation velocity profile without zooming.

**Figure 19:**
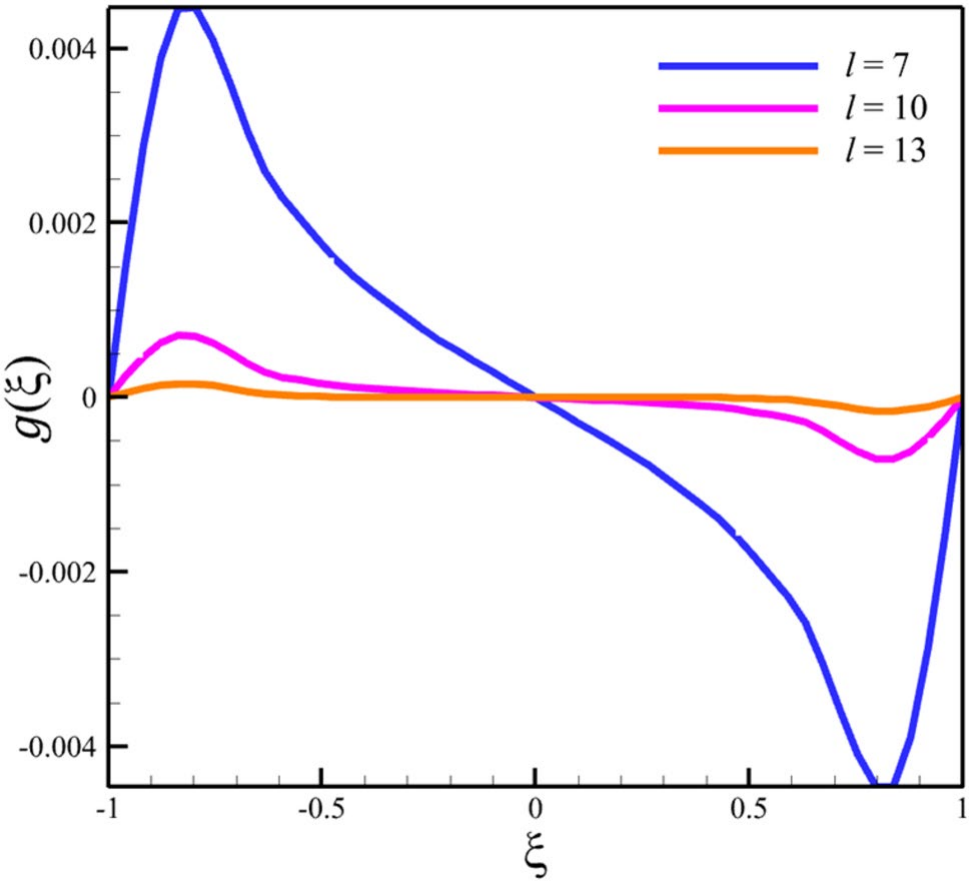
The effect of the distance between the two disks on the micro-rotation velocity profile with zooming.

**Figure 20:**
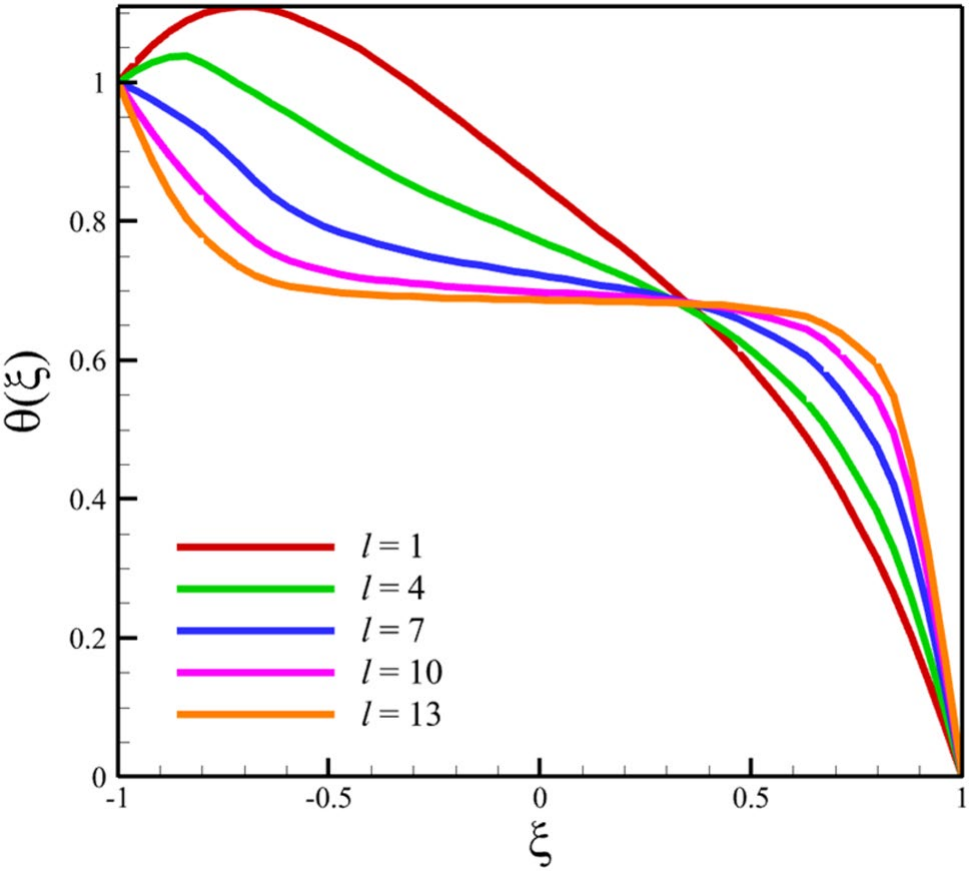
The effect of the distance between the two disks on the temperature profile.

**Figure 21:**
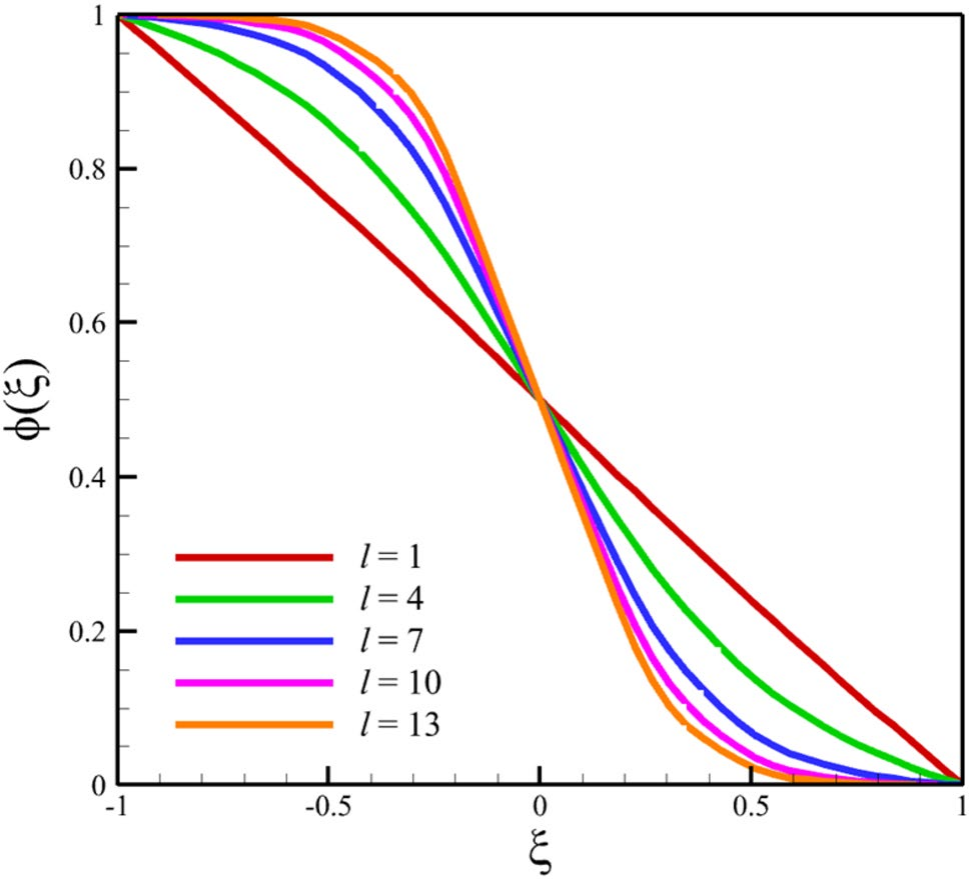
The effect of the distance between the two disks on the concentration profile.

**Figure 22:**
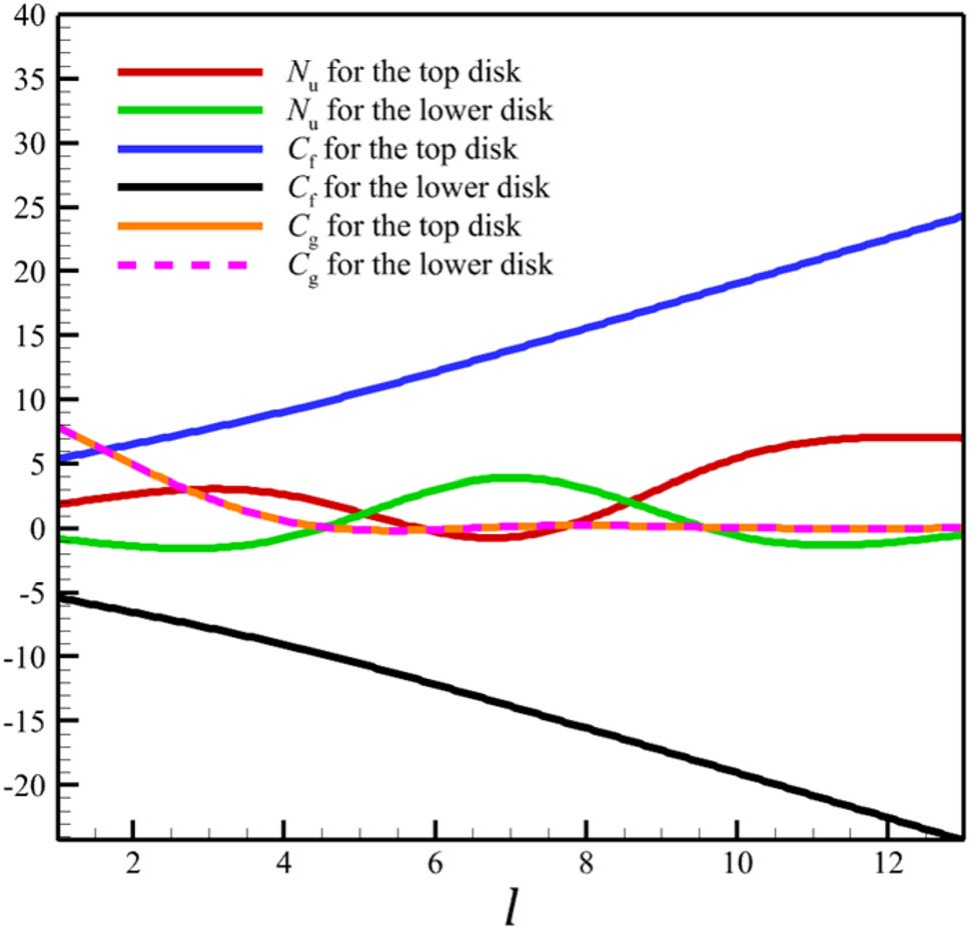
The variations of the Nusselt number (${N}_{\mathrm{u}}$), skin friction coefficient (${C}_{\mathrm{f}}$), and wall couple stress (${C}_{\mathrm{g}}$) with increasing the distance between the two disks.

**Figure 23:**
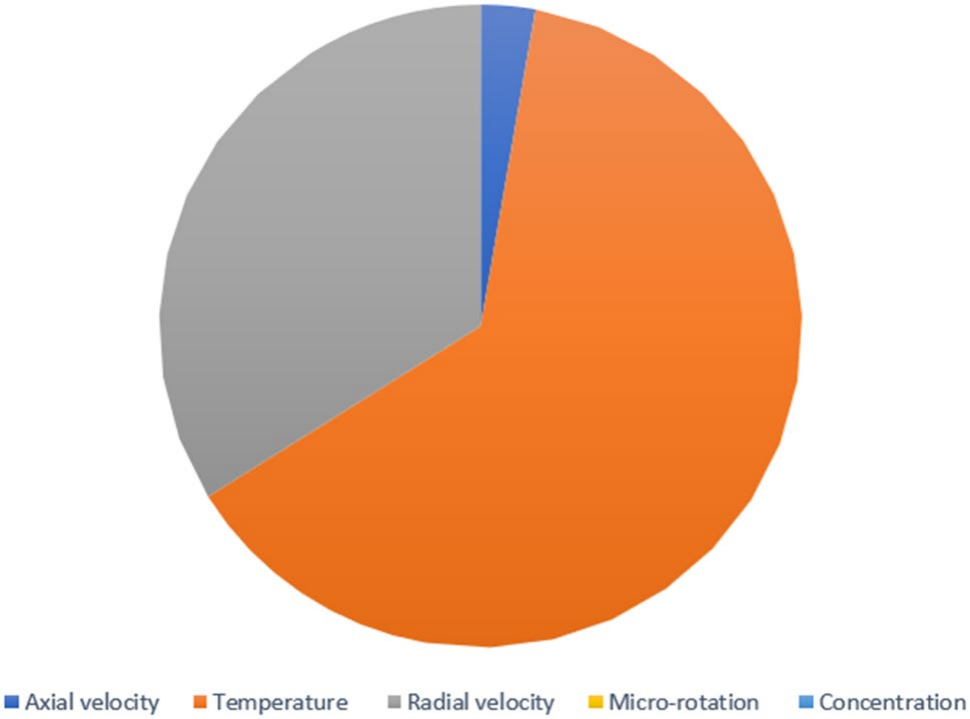
The contribution of the increase of each quantity by increasing the distance between the two disks when the value of $l$ changes from 1 to 13 and $S=1$.

Among the structural parameters, when the distance between the two disks changes, the micro-rotation velocity profile will change the most, while the profiles of axial and radial velocities, temperature, and concentration do not change significantly when the distance between the two disks changes. This high sensitivity of the micro-rotation velocity profile to the distance between the two disks is shown in Figs. 18, 19. That is, when the parameter $l$ changes from 1 to 13 and $S=1$, the micro-rotation velocity quantity changes significantly, according to Figs. 16, 17, 20, 21, the quantities of axial velocity, microrotation, radial, and concentration change significantly when the value of $l$ changes from 1 to 13 and $S=1$. In Fig. 16, the maximum positive axial velocity decreases from 0.363720785907 to 0.132657417864 when the distance between the two disks increases from 1 to 13. In Fig. 16, the maximum negative axial velocity decreases from − 0.363720785907 to − 0.132657417864 when the distance between the two disks increases from 1 to 13. As the distance between the two disks increases, the location where positive axial velocity occurs gets closer to the top disk, and the location where negative axial velocity occurs gets closer to the bottom disk. In Fig. 17, the maximum positive radial velocity decreases from 1.04587288197 to 0.190241674735 when the distance between the two disks increases from 1 to 13. According to Fig. 18, increasing the distance between the two discs strongly affects the quantity of microrotation velocity. It strongly reduces this quantity, and for $l>4$, it was observed that the value of this quantity is almost zero. In Fig. 19, we can also see a sharp decrease in this quantity for $l>4$. According to Fig. 20, unlike the sharp increase in temperature due to the increase in the rate of stretching of the two disks, the increase in the distance between the two disks has a relatively weak effect on the temperature. Just like the effect that increasing the stretching rate of the two disks has on the concentration profile (see Fig. 11), increasing the distance between the two disks also reduced the changes in concentration near the two disks (see Fig. 21). In Fig. 22 showed that the sensitivity of microrotation is higher than all other quantities. As the distance between two disks increases, the microrotation decreases sharply and reaches almost zero. According to the statistical analysis strategy stated in the investigation of the increase in the amount of stretching of the two discs, we also perform another statistical analysis in the condition that the distance between the two discs increases. According to Fig. 16, when the distance between two disks increases from 1 to 13, the absolute value of the average axial velocity drops from 0.004314092 to 2.51516E-14. The absolute value of the difference between these two axial velocities is 0.004314092. According to Fig. 17, when the distance between two disks increases from 1 to 13, the absolute value of the average radial velocity grows from 0.198230149 to 0.251775448. The absolute value of the difference between these two radial velocities is 0.053545299. According to Fig. 18, when the distance between two disks increases from 1 to 13, the absolute value of the average microrotation drops from 6.75167E-13 to 9.26448E-14. The absolute value of the difference between these two micronations is 5.82522E-13. According to Fig. 20, when the distance between two disks increases from 1 to 13, the absolute value of the average temperature drops from 0.752763232 to 0.652687022. The absolute value of the difference between these two temperatures is 0.10007621. Meanwhile, according to Fig. 21, the increase in the distance between the two disks did not greatly affect the absolute value of the average concentration, and almost the contribution of the concentration changes due to the increase in the distance between the two disks is negligible. Therefore, according to the statistical analysis we performed, the contribution of the absolute value of the average quantities is shown in Fig. 23.

## Conclusion

### Summary of results

This study examines how an electrically conducting fluid flows in the presence of a magnetic field between two disks being stretched apart. When the distance between or the rate of stretching of the disks changes, the behavior of the fluid also changes. To investigate this, the researchers used a set of equations that describe the fluid’s behavior. They found four sets of quantities that describe the fluid’s behavior by simplifying these equations. The researchers also found nine other numbers that describe the fluid’s behavior as it interacts with the vertical magnetic field and the two stretchable disks. So far, similarity solution strategies have focused mostly on how changes in these numbers affected the fluid’s quantities. However, in this study, we only focused on physical parameters that depend on the distance between or the disks’ stretching rate. By doing so, we redefined some of the numbers in the equations to better describe these physical parameters. This redefinition is the main new and novel contribution of the study. The results of changing structural parameters in this study are derived from the HAN solution, and a summary of the results in qualitative form is concluded as follows:

- Changing both structural parameters will affect all five radial and axial velocities quantities, micro-rotation, temperature, and concentration.
- Variation in the stretching rate of two disks strongly affects the temperature profile.
- The temperature profile will increase extremely as the stretching rate increases.
- Variation of the distance between the two disks strongly affects the micro-rotation velocity.
- The micro-rotation velocity will decrease extremely as the distance between the two disks increases.

### For further study

According to the assumptions of this study, only structural parameters such as $l$ and $S$ are investigated; however, investigating the impact of entropy generation or investigating the mentioned parameters simultaneously is far from the aims of current study but is recommended for further works.

## Data Availability

The datasets used and/or analysed during the current study available from the corresponding author on reasonable request.

## Abbreviations

The HAN method: The Hybrid Analytical and Numerical method
$r,\theta ,z$: Circular cylindrical coordinate system ($\mathrm{m}$, $\mathrm{rad}$, $\mathrm{m}$)
$u, w, {N}_{2}$: Fluid velocity components ($\mathrm{m}/\mathrm{s}$)
$\mathrm{U}$: Local flow velocity of the continuum ($\mathrm{m}/\mathrm{s}$)
$l$: Distance from $r$-axis ($\mathrm{m}$)
$$p$$: Pressure field ($$\mathrm{Pa}$$)
$$\mu $$: Dynamic viscosity ($$\mathrm{kg}/\mathrm{m s}$$)
$$\kappa $$: Vortex viscosity
$j$: Micro-inertia per unit mass
${{N}_{1},N}_{2},{N}_{3}$: Components of the microrotation ($\mathrm{m}/\mathrm{s}$)
$$\rho $$: Fluid density ($$\mathrm{kg}/{\mathrm{m}}^{3}$$)
${\alpha }_{1},{\alpha }_{2},{\alpha }_{3}$: Gyro-viscosity coefficients
$T$: Temperature scalar field ($\mathrm{K}$)
${\sigma }_{\mathrm{el}}$: Electrical conductivity of the fluid ($\mathrm{S}/\mathrm{m}$)
${T}_{1}$: Temperature of the lower disk ($\mathrm{K}$)
${T}_{2}$: Temperature of the upper disk ($\mathrm{K}$)
$C$: Concentration scalar field ($\mathrm{mol}/{\mathrm{m}}^{3}$)
${C}_{1}$: Concentration of the lower disk ($\mathrm{mol}/{\mathrm{m}}^{3}$)
${C}_{2}$: Concentration of the upper disk ($\mathrm{mol}/{\mathrm{m}}^{3}$)
$$\xi $$: Similarity variable (−)
${c}_{\mathrm{p}}$: Specific heat capacity at constant pressure ($\mathrm{J}/\mathrm{kg K}$)
${q}_{\mathrm{rh}}$: Radiant heat flux ($\mathrm{J}/\mathrm{s}$)
${B}_{\mathrm{os}}$: Strength of the magnetic field ($\mathrm{A}/\mathrm{m}$)
${C}_{\mathrm{f}}$: Skin friction coefficient (−)
${C}_{\mathrm{g}}$: Wall couple stress (−)
$\sigma$: Stefan-Boltzmann constant ($\mathrm{W}/{\mathrm{m}}^{2} {\mathrm{K}}^{4}$)
${k}_{a}$: Average absorption coefficient (${\mathrm{cm}}^{-1}$)
$S$: Stretching parameter of the disks (${\mathrm{s}}^{-1}$)
$$\theta (\xi )$$: Dimensionless temperature (−)
$$\phi \left(\xi \right)$$: Dimensionless concentration (−)
$f(\xi )$: Dimensionless axial velocity (−)
$f\mathrm{^{\prime}} (\xi )$: Dimensionless radial velocity (−)
$g(\xi )$: Dimensionless micro-rotation velocity (−)
${R}_{0}$: Stretching Reynolds number (−)
$${M}_{\mathrm{n}}$$: Magnetic parameter (−)
$${\lambda }_{1}$$: Vortex viscosity (−)
${\lambda }_{2}$: Micro-inertial density (−)
${\lambda }_{3}$: Spin gradient viscosity parameter (−)
$${P}_{\mathrm{r}}$$: Prandtl number (−)
$${N}_{\mathrm{r}}$$: Radiation parameter
$${E}_{\mathrm{c}}$$: Eckert number (−)
$$Sc$$: Schmidt number (−)
$r$: Radius of the disk ($\mathrm{m}$)
${R}_{\mathrm{e}}$: Local Reynolds number (−)
$${N}_{\mathrm{u}}$$: Nusselt number (−)
$k$: Thermal conductivity coefficient ($\mathrm{W}/\mathrm{m K}$)
${D}_{\mathrm{e}}$: Diffusion coefficient/mass diffusivity (${\mathrm{m}}^{2}/\mathrm{s}$)
$\Phi$: Viscous dissipation function ($\mathrm{W}/\mathrm{s }{\mathrm{kg}}^{-1} {\mathrm{m}}^{-2}$)
$F$: The name of a ordinary differential equation
$G$: The name of a ordinary differential equation
$\Theta$: The name of a ordinary differential equation
$\Phi$: The name of a ordinary differential equation
